# Disseminated Gonococcal Infection Presenting as Isolated Septic Arthritis: A Case Report and Review of Literature

**DOI:** 10.7759/cureus.66071

**Published:** 2024-08-03

**Authors:** John M Sousou, Jay Talati, Christopher J Mikulas, Ansley T Grogan, James E Farah, Pramod Reddy

**Affiliations:** 1 Internal Medicine, University of Florida College of Medicine – Jacksonville, Jacksonville, USA; 2 Medicine, University of Florida College of Medicine, Gainesville, USA; 3 Radiology, University of Florida College of Medicine – Jacksonville, Jacksonville, USA

**Keywords:** neisseria gonorrhoeae, polyarthralgia, septic arthritis, gonococcal bacteremia, disseminated gonorrhea, gonorrhea

## Abstract

*Neisseria gonorrhoeae* is a gram-negative diplococcus that passes from one person to another through sexual contact. On rare occasions, *Neisseria gonorrhoeae *may spread from the primary mucosal site to distant parts of the body and present with signs of systemic illness; this is commonly known as disseminated gonococcal infection (DGI). We present a case report of an 18-year-old patient who was diagnosed with septic arthritis of the right third metacarpophalangeal (MCP) joint without mucosal involvement or systemic symptoms and who was found to have gonococcal bacteremia. This case highlights the importance of clinician awareness of the many extragenital manifestations of DGI and a high index of suspicion in the setting of septic arthritis and high-risk sexual practices. Diagnosing DGI early and providing prompt treatment may prevent complications of sepsis, joint destruction, and a prolonged hospital stay.

## Introduction

*Neisseria gonorrhoeae* is a gram-negative diplococcus that passes from one person to another through sexual contact [1]. In 2020, the WHO estimated 82.4 million new infections of *Neisseria gonorrhoeae* among adults aged 15-49 years old. In the United States, gonorrhea remains the second most commonly reported sexually transmitted infection [2]. *Neisseria gonorrhoeae* infection starts with the adhesion of gonococci to epithelial cells through pili and surface proteins and is followed by rapid multiplication. It can induce localized infection at the site of inoculation, which is typically mucosal surfaces such as the urethra, cervix, pharynx, anus, and conjunctiva [3]. Some patients remain asymptomatic while others may have dysuria, vaginal discharge, and abdominal pain. Common complications that may arise include pelvic inflammatory disease, ectopic pregnancy, salpingitis, and infertility [4]. On rare occasions, this bacteria may disseminate into the bloodstream and manifest systemically as fever, tenosynovitis, skin lesions, septicemia, migratory polyarthritis, and vasculitis. This is known as disseminated gonococcal infection (DGI) and is seen in approximately 0.5-3% of all patients with *Neisseria gonorrhoeae* [5]. 

DGI typically presents in patients with two clinical forms. The most common form involves a triad of arthritis, dermatitis, and tenosynovitis. The second less common form is purulent arthritis, which is an abrupt onset of asymmetric pain and swelling of distal joints, and patients are often afebrile [6]. Complications that may occur as a result of DGI include endocarditis, meningitis, myositis, joint destruction, and osteomyelitis [7]. DGI typically develops within days or weeks following primary infection of a mucosal site [8]. Diagnosis is made commonly by nucleic acid amplification (NAA) tests and culture specimens from mucosal sites (urogenital, pharyngeal, or rectal) and disseminated sites (blood, cerebrospinal fluid, skin, or synovial fluids) [6]. 

We present the case of an 18-year-old female who was admitted to the hospital for septic arthritis of the right third metacarpophalangeal (MCP) joint without mucosal involvement or systemic symptoms, complicated by DGI.

## Case presentation

**Figure 1:**
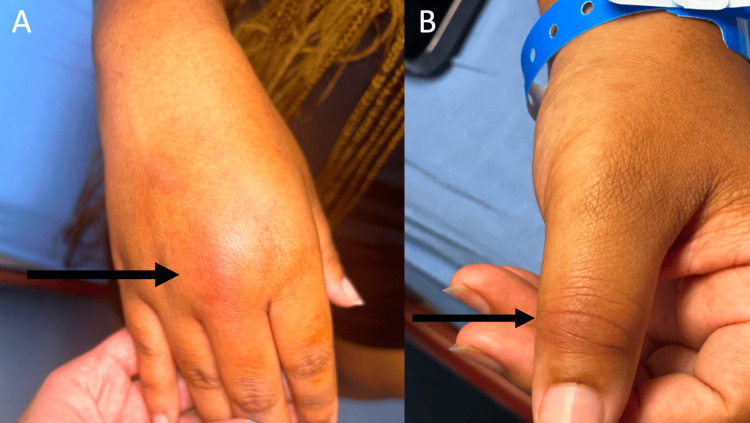
Significant erythema and swelling surrounding the right third MCP joint (A) and left IP joint of the thumb (B). MCP: metacarpophalangeal; IP: interphalangeal

The patient is an 18-year-old African American female with no significant past medical history who presented to the emergency department with right dorsal hand pain and swelling, left thumb pain, and bilateral ankle pain which started two days prior to admission. She described her pain as constant and exacerbated by movement of the associated joints, especially the dorsum of her right hand around the third MCP joint. She reported pain on the dorsum of her bilateral feet while walking. She denied any fevers, chills, recent infections, dysuria, numbness, tingling, vision changes, skin rashes, bug bites, or trauma to the hand. She denies a family history of autoimmune disease. She reported sexual activity with one male partner over the last year and denied the use of barrier contraception. A physical exam revealed significant swelling, erythema, tenderness to palpation, and an inability to flex the digits of the third MCP joint (Figure 1A). In addition, the patient had erythema and tenderness to palpation of the left interphalangeal (IP) joint of her thumb (Figure 1B).

**Figure 2:**
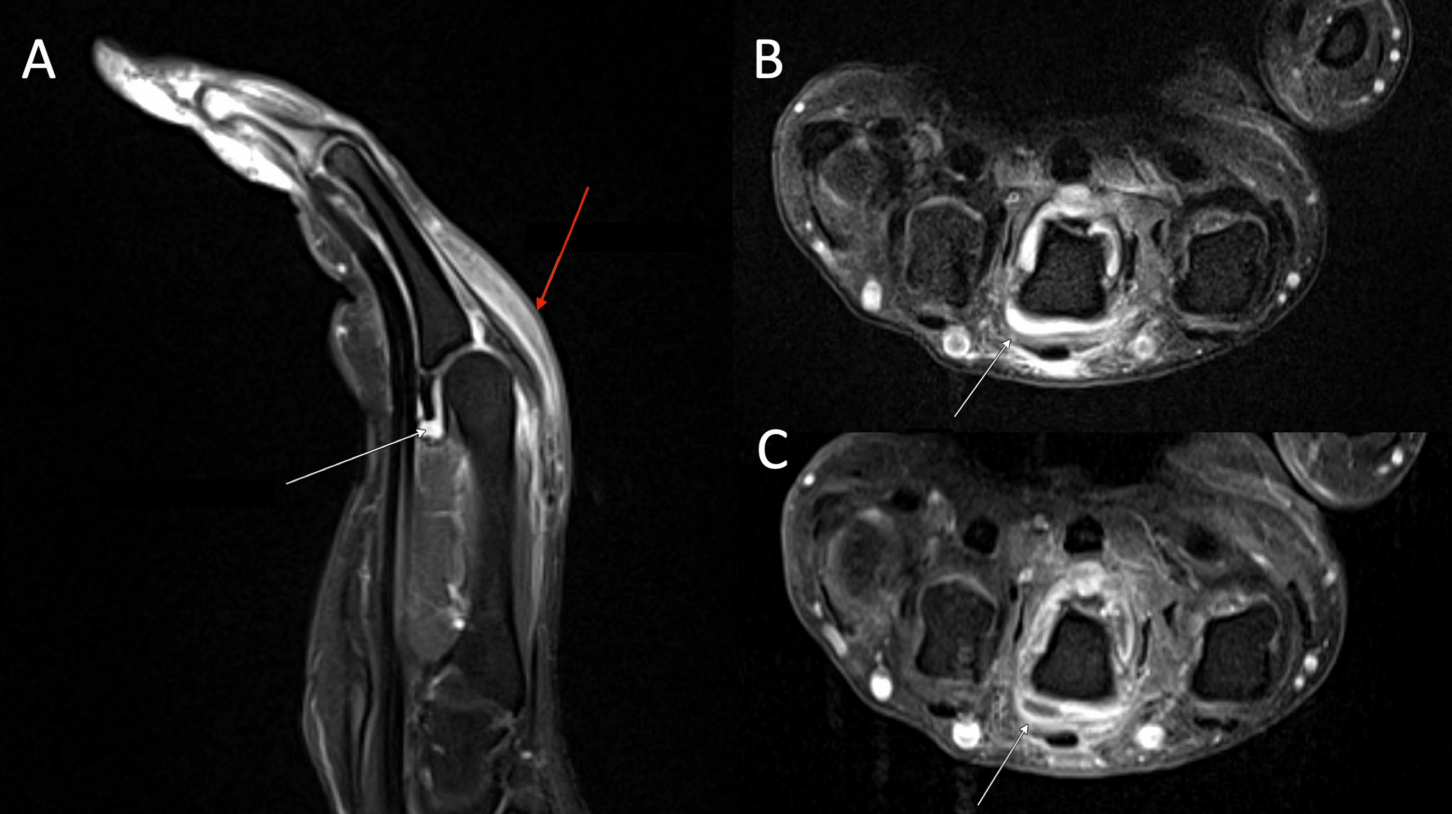
MRI of the right hand showing T1-weighted sagittal (A), proton density with contrast (B), and T1-weighted post-contrast (C) views showing synovial enhancement, effusion, and periarticular edema surrounding the third right MCP joint. (A) Joint capsule hyperenhancement is indicated by the white arrow, and subcutaneous hyperenhancement is indicated by the red arrow. (B) Synovial hyperenhancement is highlighted by the white arrow. (C) Effusion is shown by the white arrow. MRI: magnetic resonance imaging; MCP: metacarpophalangeal

Laboratory values on arrival were notable for leukocytosis of 16.05 × 10^9^/liter (L), elevated erythrocyte sedimentation rate of 47 millimeters/hour (mL/hr), and C-reactive protein of 264 milligrams (mg)/L. Pregnancy testing and testing for HIV, syphilis, hepatitis, antinuclear antibody, anti-double-stranded DNA, and a respiratory viral panel were negative. A bilateral hand X-ray revealed no acute osseous abnormalities. Magnetic resonance imaging (MRI) of the right hand revealed a large third MCP joint effusion with synovial enhancement and marked surrounding periarticular edema, concerning for septic arthritis (Figure 2). Orthopedic surgery was consulted and recommended one dose of intravenous (IV) vancomycin for empiric coverage of the septic joint and took the patient to the operating room on the day of admission for arthrotomy with debridement and irrigation (D&I) of the right third MCP joint. 

Given the patient's high-risk sexual history, a urine NAA test was ordered for sexually transmitted diseases, which revealed *Neisseria gonorrhoeae* and *Trichomonas vaginalis*. Blood cultures resulted positive for *Neisseria gonorrhoeae* within 24 hours of admission. The patient was subsequently initiated on IV ceftriaxone 1 gram daily for a total of 14 days for the treatment of her DGI, along with a seven-day course of oral metronidazole 500 milligrams twice daily for *Trichomonas vaginalis*. She was discharged from the hospital with daily home health care for the administration of IV ceftriaxone and was advised to notify her sexual partner of her diagnoses so that they may be tested and treated. The patient ultimately completed her therapy and followed up with orthopedic surgery two weeks later when sutures from the procedure were removed, and she reported resolution of her joint pain and swelling.

## Discussion

DGI remains a rare but serious complication of *Neisseria gonorrhoeae* infection. The clinical presentation of DGI varies but typically manifests with arthritis, dermatitis, and tenosynovitis. The patient in our case had an atypical presentation with septic arthritis of the right third MCP joint without the classic mucosal involvement, urinary or vaginal symptoms, or systemic symptoms. The management of DGI requires prompt antimicrobial therapy and, in cases of septic arthritis, surgical intervention for D&I of the affected joint, as performed on our patient. In this case, the patient initially received empiric vancomycin, followed by ceftriaxone based on culture results, aligning with current guidelines recommending IV ceftriaxone for the treatment of DGI [2]. The concurrent diagnosis of *Trichomonas vaginalis*, identified through NAA test, was addressed with a course of metronidazole, highlighting the importance of screening for co-infections in sexually active patients.

Risk factors for DGI have predominantly included female sex, recent menstruation, pregnancy, terminal complement deficiency, and systemic lupus erythematosus [9]. Additional risk factors have arisen in recent literature including increased prevalence in men, HIV patients, drug/alcohol users, and eculizumab use [9]. From 2009 to 2017, diagnosis rates of gonorrhea in the United States have increased by 75% due to increased prevalence and screening of the disease [10]. Given an increased rate of *Neisseria gonorrhoeae* diagnoses and the potential for debilitating complications of DGI, it is crucial to take a detailed history and provide timely intervention. Additionally, educating patients about safe sexual practices and counseling them to inform their sexual partner(s) to undergo testing and treatment is crucial. 

While DGI can present more commonly in the bacteremic form that causes dermatologic lesions, tenosynovitis, and migratory polyarthralgia, DGI may also manifest as localized septic arthritis. Our patient presented with septic arthritis which is thought to arise from bacteremic spread to the joint space after initial sexual acquisition of the infection. Our patient lacked typical features of a urinary tract infection (dysuria, frequency, and urgency) and also tested negative the week prior to the hospital admission for gonorrhea. The initial inoculation of *Neisseria gonorrhoeae* may be asymptomatic in over 50% of infections [11]. Hence, it is important to be cognizant of the various extragenital manifestations of DGI. Although any joints may be susceptible to septic arthritis in the setting of DGI, larger joints such as the knees, wrists, and ankles are more commonly affected [12]. Sexually active patients who present with localized septic arthritis should have DGI in their differential diagnosis as gram stain and synovial fluid cultures may remain positive in fewer than 50% of cases and blood cultures are found to be positive predominantly in the arthritis-dermatitis form [12]. Interdisciplinary communication between the primary hospital provider, surgeon, and pathologist can help enhance diagnostic yield and prevent a prolonged and complicated hospital course.

## Conclusions

This case highlights the importance of considering DGI as a differential diagnosis in sexually active patients presenting with septic arthritis, even in the absence of the characteristic mucosal and systemic symptoms. Early diagnosis and prompt antimicrobial treatment are critical in preventing severe complications, such as joint destruction and septic shock. Continued education on safe sexual practices and the importance of partner notification and treatment are essential components in managing and preventing the spread of *Neisseria gonorrhoeae*.
